# Key challenges to voluntary medical male circumcision uptake in traditionally circumcising settings of Machinga district in Malawi

**DOI:** 10.1186/s12889-021-11979-z

**Published:** 2021-10-28

**Authors:** Rodney Masese, Gertrude Mwalabu, Pammla Petrucka, Patrick Mapulanga

**Affiliations:** 1 Department of Community Health, University of Malawi, Kamuzu University of Health Sciences, Private Bag 1, Lilongwe, Malawi; 2 Medical-Surgical Department, Kamuzu University of Health Sciences, Private Bag 1, Lilongwe, Malawi; 3 College of Nursing, University of Saskatchewan, 119 4400 - 4th Avenue, Regina, Saskatoon, Canada; 4 Library Department, Kamuzu University of Health Sciences, Private Bag 1, Lilongwe, Malawi

**Keywords:** Challenges, Malawi, VMMC uptake, Traditional circumcision, Cultural significance

## Abstract

**Background:**

Voluntary medical male circumcision (VMMC) is becoming more popular as an important HIV prevention strategy. Malawi, with a high HIV and AIDS prevalence rate of 8.8% and a low male circumcision prevalence rate of 28% in 2016, is one of the priority countries recommended for VMMC scale-up. This paper investigates the attitudes and key challenges to VMMC adoption in a traditionally circumcising community in Malawi where male circumcision is culturally significant.

**Methods:**

A mixed design study using quantitative and qualitative data collection methods was carried out to determine the attitudes of 262 randomly selected males towards VMMC in a culturally circumcising community in Malawi. Statistical Package for the Social Sciences (SPSS) version 20 was used to analyse the quantitative data. To identify predictors of VMMC uptake, we used logistic regression analysis. To identify the themes, qualitative data were analysed using content analysis.

**Results:**

The findings indicate that, while more males in this community prefer medical circumcision, traditional circumcision is still practised. Panic (63%) perceived surgical complications (31%), and cost (27%) in accessing VMMC services were some of the barriers to VMMC uptake. Age and culture were found to be statistically significant predictors of voluntary medical male circumcision in the logistic analysis. According to qualitative data analysis, the key challenges to VMMC uptake were the involvement of female health workers in the circumcision team and the incentives provided to traditional circumcisers.

**Conclusion:**

According to the findings of this study, VMMC services should be provided in a culturally competent manner that respects and considers existing cultural beliefs and practices in the community. Coordination between local leaders and health workers should be encouraged so that VMMC services are provided in traditional settings, allowing for safe outcomes, and increasing VMMC uptake.

## Background

In recent years, voluntary medical male circumcision (VMMC) has emerged as an effective and cost-effective intervention for reducing the risk of heterosexual transmission of HIV and other Sexually Transmitted Infections [1]. This follows the findings of three randomised control studies conducted in South Africa, Kenya, and Uganda between 2005 and 2007, which found a 60% relative risk reduction in female-to-male HIV transmission among circumcised men [2–4]. As a result, the World Health Organization (WHO) and the Joint United Nations Programme on HIV/AIDS (UNAIDS) recommended VMMC as part of a comprehensive HIV prevention package in countries with high HIV prevalence and low male circumcision rates in 2007 [5]. In Malawi, however, the national voluntary medical male circumcision program was formally launched in 2012, with the National Policy on VMMC endorsing male circumcision as a core HIV prevention intervention [6].

Malawi is one of the 14 priority countries with the highest-burden recommended to expand VMMC services as an HIV prevention strategy, with an estimated 8.8%HIV prevalence rate among those aged 15–49 and nearly 70,000 new infections each year [7, 8]. Despite this recommendation, its implementation remains extremely low, particularly among traditionally circumcised communities [9]. Male circumcision, like in other African communities, is done for religious or cultural reasons rather than for medical reasons [10–12].

Traditional circumcision is highly valued as an opportunity for young men to be educated in a secluded location about manhood-related life skills for them to develop into men [12]. The young initiates are kept away from the public for up to 4 weeks while circumcised men teach them things like respect for elders, how to treat women, and how to protect and care for one’s family [12]. While medical male circumcision is not regarded as prestigious due to the use of anaesthetics to suppress pain, those who are either uncircumcised or circumcised at health facilities are mocked for not being circumcised traditionally [13]. Several other studies [12] have found strong support for traditional male circumcision in many African communities for a variety of reasons.

Even though many people in traditionally circumcising communities prefer VMMC, the practice has not changed because of social pressure from the community on traditional circumcision [14]. Human resource constraints due to a lack of trained health personnel to perform VMMC, as well as a scarcity of supplies, have been identified as factors contributing to the continued practice of traditional male circumcision [12, 15]. This has not only hampered priority countries’ efforts to scale up VMMC, but it has also harmed service quality and safety [5].

Cultural beliefs and practices, on the other hand, continue to be major motivators for traditional circumcision in Africa. For example, in a traditionally circumcising community in South Africa, 70% of young men were compelled to undergo traditional circumcision due to fear of being stigmatized for undergoing medical circumcision [13]. Similarly, the majority (85.4%) of circumcisions in Malawi, particularly where the study was conducted, were traditional [16], which is associated with risky practices that could transmit HIV. Another study found that more traditionally circumcised men (12%) were HIV-positive compared to uncircumcised men (10%) due to harmful practices such as ritual sex performed after circumcision [17].

In many African communities, ritual sex is a practice associated with traditional circumcision in which men are “cleansed through” and/or “trained on” sexual activities. It has been reported that circumcised men who have had ritual sex are twice as likely as circumcised men who have never had ritual sex to be HIV positive [17]. Traditional circumcision has been linked to similar reports of sexually risky behaviour, such as having multiple sexual partners and the misconception that HIV-positive men who are circumcised cannot transmit the virus [14, 18, 19]. VMMC has been widely advocated for scale-up to replace the risky practices associated with traditional male circumcision to prevent further HIV transmission.

Evidence suggests that large-scale implementation of medical male circumcision is required to achieve a significant reduction in HIV [1]. This meant medically circumcising 80% of Malawi’s adult males within 5 years or reaching approximately 2.1 million HIV-negative adult males by 2015 [20]. In light of this, the Malawian government developed and implemented a national policy on VMMC to expand and encourage males aged 15 to 49 who had not previously been circumcised to undergo medical circumcision [20]. By the end of 2014, however, only 150,000 male circumcisions had been performed [21], far short of the target set for priority countries with high HIV prevalence rates and low levels of medical circumcision [22]. As a result, Malawi shifted the focus of VMMC to aim for 60% circumcision of males aged 10 to 34 by 2025, based on recent study recommendations [21]. This equates to performing 3 million additional medical circumcisions to prevent 28% of new HIV infections [23].

While many males are becoming more aware of the health benefits of medical circumcision and, as a result, prefer the service at health facilities [15], successful implementation of the VMMC program in other traditionally circumcising communities faces some challenges. In a traditional circumcision-practising South African community, most men were unwilling to undergo medical circumcision or allow their sons to do so due to religion/culture, notions of manhood, and social disapproval, even though 66% of them were aware of the preventive benefit of circumcision [11].

Although the prevalence of male circumcision in Malawi has increased from 22% in 2010 to 28% in 2015–16, many of the circumcisions are traditional, with only 9% performed by a health professional [16]. Little research has been published on the barriers to VMMC in traditionally circumcising communities, as previous studies have focused on VMMC uptake in non-circumcising communities. Nonetheless, fear of pain from the procedure, perception of low HIV risk, lack of partner support, fear of HIV testing, reluctance to abstain from sex and myths and misconceptions are commonly reported barriers to VMMC uptake in traditionally non circumcising communities [24]. Other barriers include negative perceptions of VMMC as a practice for other cultures and religions, perceptions of VMMC as ineffective [25], and economic loss due to missed work and income loss during the procedure and healing period [26]. There is a dearth of literature on the barriers to VMMC in traditionally circumcising communities and how to overcome them. As a result, this study was conducted to ascertain both the attitude and key challenges to the uptake of VMMC in this traditionally circumcising community.

## Methods

### Study design and study area

The study employed a mixed design that included both quantitative and qualitative methods, with the quantitative being a cross-sectional survey and the qualitative being a key informant and focus group discussions (FGDs). The design was chosen to confirm and supplement views from questionnaire respondents and participants that would not have been obtained using only quantitative methods [27]. The author collected both quantitative and qualitative data with the assistance of Health Surveillance Assistants (HSAs). These are community health workers who were trained prior to the study to become acquainted with the data collection instrument and the actual study. The research was carried out in Machinga District, one of Malawi’s four main traditionally circumcising districts with a population of nearly a million people [8]. The district was chosen at random from among the four circumcising districts, and study participants were identified with permission from local leaders.

### Study population and sampling

The population of the study included 7707 households from Traditional Authority Chamba and 8400 from Traditional Authority Mposa in Machinga district [16]. See Table 1 on the total population for the study.

**Table 1:** Population for the study

Traditional Authority	Male-headed household		Female-headed Households		Population	Relative Frequency
	Male	%	Female	%		%
Mposa	5480	54.5%	2920	48.2%	8400	52.2%
Chamba	4567	45.5%	3140	51.8%	7707	47.8%
Total	10,047	100%	6060	100%	16,107	100%

As a result, the total population for the study was 16,107 households. However, 6060 female-headed households were excluded from the study based on the gender attribute vis a vis male circumcision, thereby remaining with 10, 067 male-headed households. The Taro Yamane method for determining sample size was used to calculate the sample size for this study. The Taro Yamane method is mathematically illustrated below:

$$ n=\frac{N}{\left[1+N{(e)}^2\right]} $$

Where:

n signifies the sample size.

N signifies the population under study.

e signifies the margin error (set at 0.05).

The sample size was 384 as a result of this calculation. This sample size is also based on the single cross-sectional study sample calculation that quantitative researchers recommend [28]. However, in this study, 262 people completed the questionnaires, representing a 68.2% response rate. Other observations were missing from the questionnaires. Respondents were comprised of married, unmarried, circumcised and uncircumcised males. The only exception was men headed households and aged 15 to 49 and agreed to take part. This age group is also considered sexually active. Participants between the ages of 15–18 heading a household were required to obtain parental consent because they are considered minors in Malawi; those who refused were excluded.

The qualitative study’s eligible participants were purposefully sampled to obtain rich information about the phenomenon of interest [29]. Four FGDs were held, with circumcised and uncircumcised males divided into young (15–24) and old (15+) groups (25–49). The number of people who took part in the focus groups ranged from 8 to 12. Furthermore, ten key informants were chosen for in-depth interviews. The number of respondents was determined by data saturation, which occurred when additional data collection based on research questions yielded no new insights [30]. There were 58 participants for the qualitative data who were not questionnaire respondents.

### Data collection and management

We collected quantitative and qualitative data at the same time, with the help of indigenously trained community health workers (HSAs). The World Health Organization (WHO) validated, modified questionnaires and interview guides, as well as the VMMC situation analysis tool kit, were used [31]. This tool has been widely used by WHO-accredited organizations in several other VMMC studies, with high levels of reliability [9].

Pretesting was conducted among 5 respondents in a neighbouring village to ensure construct and content validity of the data collection tool before administering the final data collection tool to subjects as recommended by researchers [32]. Every third household in the randomly selected villages was chosen to identify a potential respondent.

Questionnaires were distributed to participants at their homes, while FDGs were held at a central and private location within the villages. The questionnaires were coded and entered a computer system. Prior to analysis, data cleaning was performed to check for errors in data entry.

### Statistical and content analyses

To analyse quantitative data, we used the Statistical Package for the Social Sciences (SPSS) version 20 to generate frequencies and cross-tabulations. The association between independent variables and decision making regarding voluntary medical male circumcision uptake as a dependent variable was determined using logistic regression.

### Variable selection

The first step in the logistic regression analysis was to recode the outcome variable so that it became “vmmc” (0 = traditional; 1 = vmmc). Following that, we used chi-square analysis to identify potential predictors, and all predictors with *p*-values less than or equal to 0.25 were chosen as potential predictors for the regression. In the regression, the following analytical framework was used:

$$ \log \left(\frac{P\left(Y=1\right)}{P\left(Y=0\right)}\right)={\beta}_0+{\beta}_1 age+{\beta}_2 marital\_ status+{\beta}_3 vmmc\_ hindrance $$

Where Y = VMMC, and *β*_1_, *β*_2_, and *β*_3_ are regression constants.

Except for age, we discovered that standard errors for almost all predictors were significantly inflated when compared to their corresponding estimates during preliminary regression analysis. Then, for marital status and VMMC hindrance, we created dummy variables. Following that, dummies with the highest *p*-value were removed at each stage until the standard errors were found to be relatively smaller than their corresponding estimates.

Iterative analysis of qualitative data was used to identify themes. We read the transcripts several times to familiarize ourselves with their contents [33], and then key elements in the answers were identified after classifying similar responses into themes and subthemes. Themes were created through intuition, immersion, and repeated reading, sorting, and coding of data. We extensively engaged with the participants to better understand their perspectives to ensure that the collected data was objective, accurate, dependable, and extrapolable.

Furthermore, methodological triangulation was performed to have rich data from multiple perspectives. This meant that the data collected met the credibility, dependability, conformability, transferability, and authenticity criteria proposed by qualitative research experts [32, 34]. Among the research questions were: *“What do people think it is like having male circumcision done? Do people have memories of it or not? Are there any risks related to the traditional male circumcision process? What suggestions might people have for improving the way male circumcision is done in the traditional setting? What might stop someone from using the health facility for medical circumcision?”*

## Results

### Characteristics of respondents

According to Table 2, a total of 262 people took part in the quantitative study. Nearly half of the interviewees (42%, *n* = 110) were young, ranging in age from 15 to 24 years old, with a mean age of 30.35 years. The remainder were adults aged 25 and up. The vast majority (90.8%, *n* = 238) stated that they had been circumcised at the time of the interviews. In terms of education, 62.6% (*n* = 164) of the participants completed primary school, 22.5% (*n* = 55) completed secondary school, and 14.9% (*n* = 39) had no education at all. All of the participants belonged to a religious group, with Muslims accounting for the vast majority (74%, *n* = 194) and Christians accounting for the remainder.

**Table 2:** Relationship of Demographic Variables and Male Circumcision Status (*n* = 262)

Item	Circumcised (%)	Uncircumcised (%)	Total (%)	***P***-value
**Age group of respondents**				.003
15–24 years	**109 (45.8)**	**1 (4.2)**	110 (42.0)	
25–34 years	53 (22.3)	1 (4.2)	54 (20.6)	
35–44 years	36 (15.1)	6 (25)	42 (16.0)	
45–49 years	40 (16.8)	16 (66.6)	56 (21.4)	
**Educational level**				.000
Primary	**152 (63.9)**	**12 (50.0)**	**164 (62.6)**	
Secondary	57 (23.9)	2 (8.3)	55 (22.5)	
None	29 (12.2)	10 (41.7)	39 (14.9)	
**Occupation**				.021
Schooling	47 (19.7)	1 (4.2)	48 (18.3)	
Farmer	**85 (35.7)**	**15 (62.5)**	**100 (38.2)**	
Casual labourer	65 (27.3)	2 (8.3)	67 (25.6)	
Employed	3 (1.3)	1 (4.2)	4 (1.5)	
Business	38 (16.0)	5 (20.8)	43 (16.4)	
**Religion**				.000
Christian	48 (20.2)	20 (83.3)	68 (26.0)	
Muslim	**190 (79.8)**	**4 (16.7)**	**194 (74.0)**	
**Tribe**				.000
Chewa	17 (7.1)	2 (8.3)	19 (7.3)	
Yao	**188 (79.0)**	**6 (25.0)**	**194 (74.0)**	
Tumbuka	1 (.4)	2 (8.3)	3 (1.1)	
Lhomwe	29 (12.2)	9 (37.5)	38 (14.5)	
Ngoni	3 (1.3)	5 (20.8)	8 (3.1)	
**Marital status**				.000
Single	**130 (54.6)**	**17 (70.8)**	**147 (56.1)**	
Married	94 (39.5)	1 (4.2)	95 (36.3)	
Widowed	5 (2.1)	2 (8.3)	7 (2.7)	
Divorced	9 (3.8)	4 (16.7)	13 (5.0)	

### Descriptive results

According to Table 3, the majority (97**%**, *n* = 223) of those circumcised (90.8**%**, *n* = 238) had the procedure done in a traditional setting. VMMC was performed on only 6.3**%** (*n* = 15) of those circumcised. This confirms that traditional circumcision is the norm in the study area. There was a significant relationship between respondents’ marital status and VMMC uptake (2 = 11.811, df = 3, *p* = 0.008), with unmarried males taking more.

**Table 3:** Relationship of Demographic Characteristics and VMMC uptake (*N* = 238)

Characteristic	Traditional MC (%)	VMMC (%)	Total (%)	***P***-value
**Age group of respondents**				0.008
15–24 years	**101 (45.4)**	**8 (53.6)**	**109 (45.9)**	
25–34 years	52 (23.4)	1 (6.7)	53 (22.3)	
35–44 years	35 (15.6)	1 (6.7)	36 (15.2)	
45–49 years	35 (15.6)	5 (33)	40 (16.6)	
**Education**				0 .329
None	**29 (13.0)**	**0 (.0)**	**29 (12.2)**	
Secondary	53 (23.8)	4 (26.7)	57 (23.9)	
Primary	**141 (63.2)**	**11 (73.3)**	**152 (63.9)**	
**Religion**				0.987
Muslim	**178 (79.8)**	**12 (80)**	**190 (79.8)**	
Christian	45 (20.2)	3 (20)	48 (20.2)	
**Tribe**				0.298
Tumbuka	1 (.4)	0 (.0)	1 (.4)	
Ngoni	2 (.9)	1 (6.7)	3 (1.3)	
Lhomwe	26 (11.7)	3 (20)	29 (12.2)	
Yao	**178 (79.7)**	**10 (66.6)**	**188 (79.0)**	
Chewa	16 (7.2)	1 (6.7)	17 (7.1)	
**Marital status**				0.008
Divorced	6 (2.7)	3 (20)	9 (3.8)	
Widowed	5 (2.2)	0 (.0)	5 (2.1)	
Married	89 (39.9)	5 (33.3)	94 (39.5)	
Single	**123 (55.2)**	**7 (46.7)**	**130 (54.6)**	

### Challenges to VMMC uptake

#### Accessibility, culture, and fear of complications

Table 4 lists the barriers to respondents‘adoption of VMMC. Fear of pain during the procedure (63%, *n* = 165) and bleeding (31%, *n* = 82) were the most frequently cited barriers to VMMC. Accessibility (37%, *n* = 97) and high cost (27%, *n* = 71) in terms of transportation to a free health facility or service payment at a private hospital were also major reasons given for not undergoing VMMC. Cultural practices and peer opposition were identified as barriers to VMMC services by 19% (*n* = 50) and 5% (*n* = 13) of respondents, respectively (Table 3).

**Table 4:** Distribution of Factors hindering Uptake of VMMC (*n* = 262)

Item	Value	Percentage (%)
**Hindrances for VMMC**		
Religion	8	3.1
Culture	50	19.0
Fear of complications	6	2.4
No interest	9	3.4
Peer opposition	13	5.0
Accessibility	**97**	**37.0**
Cost	71	27.0
Misconceptions	8	3.1
**Perceived negative consequences of VMMC**		
Infections	10	3.8
Impotence	5	1.9
Bleeding	82	31.1
Pain	**165**	**63.0**

Statistical analysis of potential predictors for VMMC at a *p-*value threshold of 0.25 indicated that age, marital status and VMMC hindrance could be potential predictors for VMMC uptake.

#### Significant predictors of VMMC

Table 5 shows the results of the analysis of potential predictors for VMMC at a *p*-value threshold of 0.25.

**Table 5:** Results of the final logistic regression model

Factor	Odds ratio & 95.0% C.I.	***P***-value
Intercept (α)	5.78	0.243
Age	0.59 (0.40, 0.86)	0.006
Married vs. other status	0.35 (0.07, 1.84)	0.214
Culture vs. other hindrances	4.81 (1.02, 22.68)	0.047

#### Modelling VMMC uptake

Table 5 presents results of the final logistic regression model. According to the findings in Table 5, age and culture are statistically significant predictors of voluntary medical male circumcision. In particular, the odds of VMMC uptake are 41% lower in older males than in younger males (Adj. OR = 0.59; 95% CI: 0.40, 0.86). Furthermore, the odds of VMMC uptake are five times higher in males who are hampered by culture than in those who are hampered by other factors (Adj. OR = 4.81, 95% CI: 1.02, 22.68). Being married, on the other hand, was not significantly associated with VMMC uptake at the 5% significance level (*P* = 0.214).

#### Assessing model fit

**Fig. 1:**
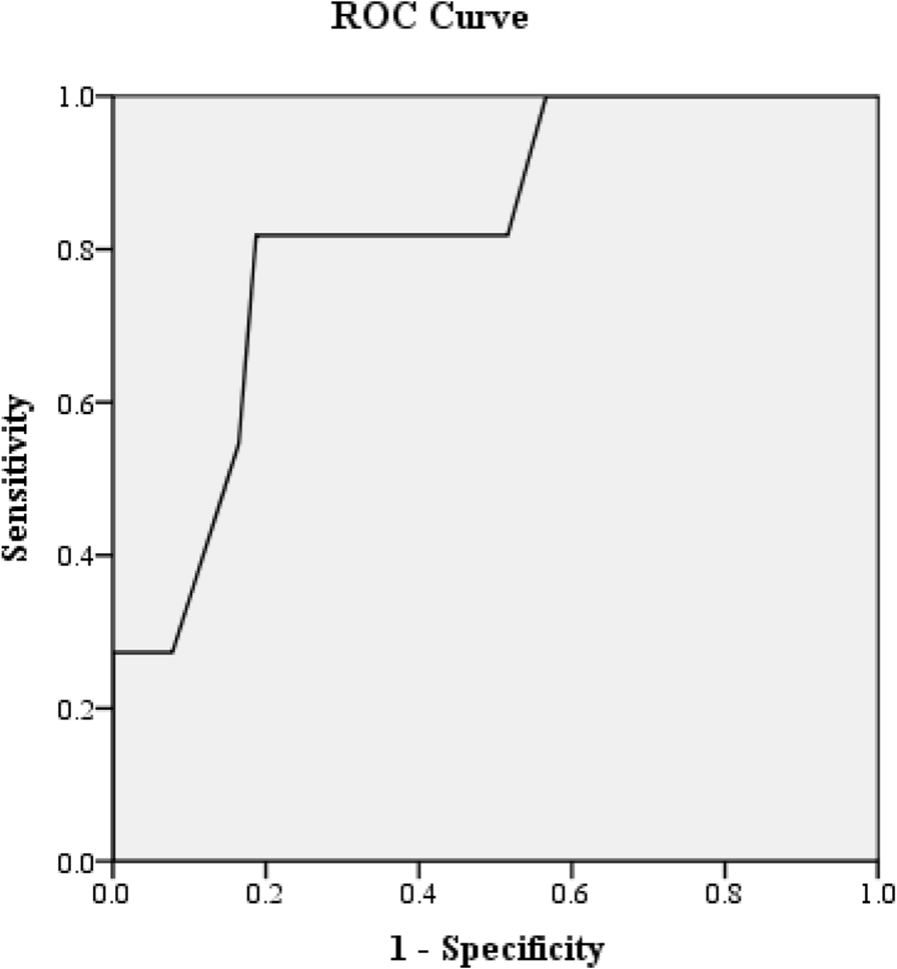
Assessment of the model fit

Figure 1 shows that the model correctly predicted VMMC uptake by a chance of 82.1% (AUC = 0.821, 95% CI: 0.707, 0.934).

Therefore, the final model is: $\log \left(\frac{P\left(Y=1\right)}{P\left(Y=0\right)}\right)=1.75-0.53 age+1.57 culture$.

#### Associating traditional circumcision with celebrations and cultural feasting

The current study’s qualitative data analysis reveals that the fun associated with traditional circumcision was a significant barrier to VMMC adoption. According to some key informants, many males are not opting for VMMC due to cultural practices such as celebrations and feasting that are often associated with traditional circumcision. One older key informant confirmed that traditional circumcision celebrations promote the traditional practice more than the VMMC:

*Here we are just used to traditional circumcision as a time for celebration and drumbeating. This brings pride in parents who prefer taking their sons to the “bush” so that they should show their expertise in celebrating. The dancing, the gifts to those who dance well, and new clothes are motivators associated with traditional circumcision* (*KI 11*).

Aside from cultural feasting, key informants frequently mentioned the financial advantages of traditional circumcision. Most informants stated that both chiefs and traditional circumcisers (Angaliba) profit financially from traditional circumcision and thus cannot promote VMMC, as stated by one key informant:

*Chiefs here are a hindrance to promoting medical circumcision since they get money for each boy who goes for traditional circumcision. Because of such a reward, it is difficult for local leaders to advocate for medical circumcision. The more traditional circumcisions are performed, the more money Angaliba and chiefs get (KI 09).*

#### Gaining financial support

During FDGs for older, married men, women’s resistance was also mentioned as a major barrier to VMMC. Most participants stated that women were largely influential in promoting traditional circumcision due to the financial benefits associated with it. It was discovered that women financially support one another for their sons to be circumcised traditionally. According to one of the older men in the group:

*Women enjoy traditional circumcisions because they gain money and gifts from their friends. If one is financially not well, they borrow each other money which is repaid with interests during initiation ceremonies, like a business. If a man insists that my son will undergo medical circumcision, you will quarrel with your wife until you fight; even the marriage would break. (FGD, Adult 04).*

#### Diluting cultural norms

Many participants explained that VMMC is seen as eroding cultural norms because male circumcision is largely a cultural phenomenon in which traditional norms are passed down through generations. During the in-depth interviews, one older participant confirmed this assertion:

*Traditional circumcision is commonly practiced here because besides being circumcised, the boys are taught good behaviour: to respect parents, elders, and to assist parents with other tasks. So, without the education that goes with traditional circumcision, where would the young males be modelled (KI 03)*

#### Being influenced by peers

Peer pressure was also identified as a significant barrier to VMMC adoption by most participants. Many participants observed that young males rush for traditional circumcision in order to fit in with their peers who are circumcised in large numbers all at once.

One participant explained it as follows:

*The young boys could not wait for medical circumcision while many of their friends are being circumcised in a traditional setting. They are afraid of being laughed at as still young by their circumcised friends! So, they all go as a group, the way they play together. They are also attracted by new clothes, dancing, and many other gifts given following traditional circumcision* (*FGD 09*).

#### Being attended to by female health providers

Participants were concerned about the lack of respect, secrecy, and presence of female health providers in the operating room during the VMMC procedure. Such female involvement was viewed as disrespectful and a complete departure from cultural norms practised at traditional circumcision camps, where older men take responsibility for caring for circumcised males until they recover completely. One of the younger participants elaborated:

*At the hospital, you find that female providers are performing circumcisions, so some feel embarrassed thinking that “if she touches my private parts I will erect since I’m not sick. It is better in the village because I will be circumcised by my fellow men so no woman would see me” (FGD Ado 10).*

## Discussion

This study identifies key barriers to VMMC adoption in Malawi’s traditionally circumcising communities. While quantitative data revealed that age, accessibility, cultural influence, and fear of post-operative complications such as pain and bleeding were all barriers to medical circumcision, qualitative data revealed more intriguing findings. The majority of participants in this study stated pain as a major impediment to VMMC, which is consistent with findings from other studies that have reported varying degrees of complications as impediments to VMMC. The most commonly reported complications of VMMC are pain, bleeding, swelling, and infection [35, 36]. Glans amputation has been reported in extremely rare cases of adverse effects [37].

However, many participants in the current study expressed fear of pain, which could be attributed to the shared traumatic experience of traditional circumcision, where pain is thought to be a prerequisite for the procedure [13]. This means that some males who had undergone traditional circumcision may have told their colleagues about the excruciating pain they felt during the cultural initiation. It is worth noting that anaesthesia is never used in such circumcisions because pain is regarded as bravery [12, 13]. As a result, interventions to increase VMMC adoption must focus on educating the public that the procedure is minor, safe, and not as painful because it is performed under local anaesthesia. Adverse events are extremely rare, as previously observed in a Tanzanian study aimed at evaluating quality in a high-volume male circumcision campaign [38].

Age and culture were found to be statistically significant predictors of voluntary medical male circumcision in the current study. During FDGs, cultural practices commonly associated with traditional circumcision, such as feasting, which is usually planned by women, were also highlighted as an important factor. Previously, women in traditionally circumcising communities were found to be supportive of medical male circumcision for their sons [12, 39], but the current study found that women preferred traditional male circumcision for their sons due to perceived cultural and financial benefits. Previous studies conducted in traditionally circumcising communities have reported benefits such as initiation into adulthood and social conformity among peers [40, 41].

Similarly, the regression model analysis revealed that marital status was not significantly associated with VMMC uptake. As a result, women were found to have no influence on their partners or sons to undergo VMMC. This highlights the importance of targeting women separately with messages about the importance of VMMC in reducing the HIV pandemic to change their attitude. Alternatively, after the initiates have undergone medical circumcision, traditional ceremonies can be encouraged. Such integration of both approaches would ensure that the perceived benefits of each approach are capitalized on.

Indeed, there is evidence that providing VMMC services in traditional settings can not only improve safety but also increase uptake and male circumcision coverage [42, 43]. However, while integrating traditional circumcision practices with modern VMMC was a successful model in Zimbabwe, more investment was required to build trust among community leaders and members [44]. Despite the fact that other studies have reported on the influential role those local leaders play in creating demand for VMMC [39, 45], the current study discovered that some local leaders and traditional circumcisers encouraged traditional circumcision due to the associated personal financial gains. This is due to the fact that for any male circumcised in a traditional setting, a monetary or non-monetary incentive is given to both the chief and the traditional circumciser (Angaliba).

As a result, the financial gain became a major motivator for traditional circumcision, and the safety of the young initiates being circumcised took precedence. Other studies [12, 46, 47] found that traditional male circumcision has a high rate of complications. This implies that, in addition to training more health workers in safe circumcision, much work needs to be done to promote behavioural change communication in order to re-pattern some of these risky cultural practices. Traditional leaders, on the other hand, can be incorporated to promote collaboration between the community and health workers in order to prevent unsafe circumcisions by untrained local providers [48].

According to the qualitative data, older males were uneasy about being attended to by female health providers in the VMMC room. This is supported by the finding that older males had a 41% lower chance of using VMMC than younger males. The presence of female health workers on the circumcision team made some males feel embarrassed, especially since they were not sick. Some men erect as a female provider prepares them for circumcision, according to participants. A study in Zambia [49] found that undressing in the presence of an opposite-sex health worker caused similar feelings of shame and embarrassment.

The fact that the majority of the participants came from a religious background where male circumcision is a sacred procedure could be the source of their discomfort. As a result, traditional male circumcision is valued as a private and sensitive practice performed in an all-male environment. This observation is supported by two studies conducted in Eastern and Southern Africa, which found that many men are uncomfortable being exposed as uncircumcised men, as well as the potential for sexual arousal [45]. This implies that VMMC services should be provided in a culturally competent manner that takes cultural beliefs into account and addresses the concerns of such men. Such considerations would entice men in traditionally circumcising communities to embrace medical circumcision rather than the risky traditional circumcision.

### Limitations

This research is based on cross-sectional data from one of Malawi’s four circumcising districts, which only allowed us to look at associations. As a result, generalizing the findings must be done with caution. Furthermore, our findings are based on self-reported circumcision status and have not been independently verified. This could have misrepresented both the participants’ circumcision status and their experiences with it. More importantly, most participants were interviewed many years after they had been circumcised, which increased the possibility of memory bias.

Furthermore, the diverse proportion of participants who were circumcised versus those who had undergone VMMC may not provide a balanced view. The sample size is the final constraint. Only 262 of the 390 total respondents answered all the questions. This may limit the non-respondents’ ability to provide representative views. We believe, however, that the use of mixed methods compensated for this limitation. Despite these limitations, this study offers policy insights into programmatic strategies for increasing VMMC uptake in traditionally circumcising communities.

## Conclusions

According to the findings of this study, VMMC uptake in traditionally circumcising communities in Malawi remains low due to cultural practices orchestrated by local leaders and women, as well as the involvement of female health workers during medical male circumcision. As a result, VMMC services should be provided in a culturally competent manner that takes traditional beliefs into account to address the concerns of such men. More health workers should be trained to provide safe circumcisions, especially in rural areas where there is a greater unmet need. Targeted public awareness campaigns should also be promoted in order to instill more positive attitudes toward VMMC and improve behavioural change communication. More importantly, the Ministry of Health, in collaboration with its partners, should lead the integration of local leaders and health workers in the scaling-up of safe circumcisions. More research is needed to determine the efficacy of an integrated VMMC model in traditionally circumcised communities.

## Data Availability

The datasets used for the current study are available from the corresponding author upon request.

## Abbreviations

AIDS: Acquired Immunodeficiency Syndrome
COMREC: College of Medicine Research and Ethics Committee
FGD: Focus Group Discussion
HSA: Health Surveillance Assistants
HIV: Human Immunodeficiency Virus
IDI: In-depth Interview
PT: Participants
SPSS: Statistical Package for the Social Sciences
TA: Traditional Authority
UNIMA: University of Malawi
VMMC: Voluntary Medical Male Circumcision
WHO: World Health Organisation

## References

[1] Cork MA, Wilson KF, Perkins S, Collison ML, Deshpande A, Eaton JW, Earl L, Haeuser E, Justman JE, Kinyoki DK, Mayala BK (2020). Mapping male circumcision for HIV prevention efforts in sub-Saharan Africa. BMC Med.

[2] Auvert B, Taljaard D, Lagarde E, Sobngwi-Tambekou J, Sitta R, Puren A (2005). Randomized, controlled intervention trial of male circumcision for reduction of HIV infection risk: the ANRS 1265 Trial. PLoS Med.

[3] Bailey RC, Moses S, Parker CB, Agot K, Maclean I, Krieger JN, Williams CF, Campbell RT, Ndinya-Achola JO (2007). Male circumcision for HIV prevention in young men in Kisumu, Kenya: a randomised controlled trial. Lancet.

[4] Gray RH, Kigozi G, Serwadda D, Makumbi F, Watya S, Nalugoda F, Kiwanuka N, Moulton LH, Chaudhary MA, Chen MZ, Sewankambo NK (2007). Male circumcision for HIV prevention in men in Rakai, Uganda: a randomised trial. Lancet.

[5] Sgaier SK, Reed JB, Thomas A, Njeuhmeli E (2014). Achieving the HIV prevention impact of voluntary medical male circumcision: lessons and challenges for managing programs. PLoS Med.

[6] Government of Malawi (2014). National HIV-AIDS Strategic Plan (2015–2020).

[7] UNAIDS W (2011). Joint strategic action framework to accelerate the scale-up of voluntary medical male circumcision for HIV prevention in eastern and southern Africa, 2012–2016.

[8] Malawi National Statistical Office (NSO) and ICF. Malawi Demographic and Health Survey 2015-16. 2017; Zomba and Rockville.

[9] Bengo J, Chalulu K, Chinkhumba J, Kazembe L, Maleta K, Masiye F, Mathanga D (2010). Situation analysis of male circumcision in Malawi.

[10] World Health Organization (2009). Traditional male circumcision among young people: a public health perspective in the context of HIV prevention.

[11] Mark D, Middelkoop K, Black S, Roux S, Fleurs L, Wood R, Bekker L (2012). Low acceptability of medical male circumcision as an HIV/AIDS prevention intervention within a south African community that practises traditional circumcision. S Afr Med J.

[12] Mshana G, Wambura M, Mwanga J, Mosha F, Changalucha J (2011). Traditional male circumcision practices among the Kurya of North-Eastern Tanzania and implications for national programmes. AIDS Care.

[13] Phega Mangena M, Mavis Mulaudzi F, Doriccah PM (2011). The experiences of nurses in caring for circumcised initiates admitted to hospital with complications. Contemp Nurse.

[14] Maughan-Brown B, Venkataramani AS, Nattrass N, Seekings J (2011). Whiteside a.W. a cut above the rest: traditional male circumcision and HIV risk among Xhosa men in Cape Town, South Africa. J Acquir Immune Defic Syndr.

[15] Wambura M, Mwanga JR, Mosha JF, Mshana G, Mosha F, Changalucha J. Acceptability of medical male circumcision in the traditionally circumcising communities in Northern Tanzania. BMC Public Health. 2011;11(373). 10.1186/1471-2458-11-373. 10.1186/1471-2458-11-373 PMC3112418 21605433

[16] National Statistical Office (NSO) [Malawi] and ICF. 2017. Malawi Demographic and Health Survey 2015-16. Zomba and Rockville. NSO and ICF.

[17] Mutombo N, Maina B, Jamali M (2015). Male circumcision and HIV infection among sexually active men in Malawi. BMC Public Health.

[18] Shi C, Li M, Dushoff J (2020). Traditional male circumcision is associated with sexual risk behaviors in sub-Saharan countries prioritized for male circumcision. AIDS Behav.

[19] Chatsika ZJ, Kumitawa A, Samuel V, Azizi SC, Jumbe VC (2020). Voluntary medical male circumcision and sexual practices among sexually active circumcised men in Mzuzu, Malawi: a cross-sectional study. BMC Public Health.

[20] Ministry of Health (2012). National policy on voluntary medical male circumcision.

[21] Government of Malawi, National AIDS Commission (2014). National strategic plan for HIV and AIDS, 2015–2020.

[22] UNAIDS (2015). World AIDS day 2015 report: On the Fast-Track to end AIDS.

[23] Njeuhmeli E, Forsythe S, Reed J, Opuni M, Bollinger L, Heard N, Castor D, Stover J, Farley T, Menon V, Hankins C (2011). Voluntary medical male circumcision: modeling the impact and cost of expanding male circumcision for HIV prevention in eastern and southern Africa. PLoS Med.

[24] Hatzold K, Mavhu W, Jasi P, Chatora K, Cowan FM, Taruberekera N, Mugurungi O, Ahanda K, Njeuhmeli E (2014). Barriers and motivators to voluntary medical male circumcision uptake among different age groups of men in Zimbabwe: results from a mixed methods study. PLoS One.

[25] Carrasco MA, Wilkinson J, Kasdan B, Fleming P (2019). Systematic review of barriers and facilitators to voluntary medical male circumcision in priority countries and programmatic implications for service uptake. Global Public Health.

[26] Evens E, Lanham M, Hart C, Loolpapit M, Oguma I, Obiero W (2014). Identifying and addressing barriers to uptake of voluntary medical male circumcision in Nyanza, Kenya among men 18–35: a qualitative study. PLoS One.

[27] Creswell J (2003). Research design: qualitative, quantitative and mixed methods approaches.

[28] Gorstein J, Sullivan KM, Parvanta I, Begin F (2007). Indicators and methods for cross-sectional surveys of vitamin and mineral status of populations.

[29] Palinkas LA, Horwitz SM, Green CA, Wisdom JP, Duan N, Hoagwood K (2015). Purposeful sampling for qualitative data collection and analysis in mixed method implementation research. Adm Policy Ment Health Ment Health Serv Res.

[30] Saunders B, Sim J, Kingstone T, Baker S, Waterfield J, Bartlam B, Burroughs H, Jinks C (2018). Saturation in qualitative research: exploring its conceptualization and operationalization. Qual Quant.

[31] World Health Organisation. WHO | Male circumcision situation analysis toolkit: WHO; World Health Organization; 2009. https://www.who.int/hiv/pub/malecircumcision/sit_analysis/en/

[32] Polit DF, Beck CT (2008). Nursing Research: Generating and Assessing Evidence for Nursing Practice.

[33] Nxumalo CT, Mchunu GG (2020). Zulu Men’s conceptions, understanding, and experiences of voluntary medical male circumcision in KwaZulu-Natal, South Africa. Am J Mens Health.

[34] Elo S, Kääriäinen M, Kanste O, Pölkki T, Utriainen K, Kyngäs H (2014). Qualitative content analysis: a focus on trustworthiness. SAGE Open.

[35] Bochner AF, Feldacker C, Makunike B, Holec M, Murenje V, Stepaniak A, Xaba S, Balachandra S, Tshimanga M, Chitimbire VT, Barnhart S (2017). Adverse event profile of a mature voluntary medical male circumcision programme performing PrePex and surgical procedures in Zimbabwe. J Int AIDS Soc.

[36] Mavhu W, Hatzold K, Dam KH, Kaufman MR, Patel EU, Van Lith LM, Kahabuka C, Marcell AV, Mahlasela L, Njeuhmeli E, Seifert AK (2018). Adolescent wound-care self-efficacy and practices after voluntary medical male circumcision—a multicountry assessment. Clin Infect Dis.

[37] Manentsa M, Mukudu H, Koloane N, Ringane A, Matta E, Martinson NA, Lebina L (2019). Complications of high volume circumcision: glans amputation in adolescents; a case report. BMC Urol.

[38] Mahler HR, Kileo B, Curran K, Plotkin M, Adamu T, Hellar A, Koshuma S, Nyabenda S, Machaku M, Lukobo-Durrell M, Castor D, Njeuhmeli E, Fimbo B (2011). Voluntary medical male circumcision: matching demand and supply with quality and efficiency in a high-volume campaign in Iringa region, Tanzania. PLoS Med.

[39] Siegler AJ, Mbwambo JK, DiClemente RJ (2012). Acceptability of medical male circumcision and improved instrument sanitation among a traditionally circumcising group in East Africa. AIDS Behav.

[40] Kioli FN, Were AR, Onkware K (2012). Traditional perspectives and control mechanisms of adolescent sexual behaviour in Kenya. Int J Sociol Anthropol.

[41] WHO (2009). Traditional Male Circumcision among Young People: A Public HealthPerspective in the Context of HIV Prevention.

[42] Bulled N, Green EC (2016). Making voluntary medical male circumcision a viable HIV prevention strategy in high-prevalence countries by engaging the traditional sector. Crit Public Health.

[43] Shumba K, Lubombo M (2017). Cultural competence: a framework for promoting voluntary medical male circumcision among VaRemba communities in Zimbabwe. Afr J AIDS Res.

[44] Hove J, Masimba L, Murenje V, Nyadundu S, Musayerenge B, Xaba S, Nachipo B, Chitimbire V, Makunike B, Holec M, Chinyoka T (2019). Incorporating voluntary medical male circumcision into traditional circumcision contexts: experiences of a local consortium in Zimbabwe collaborating with an ethnic group. Glob Health Sci Pract.

[45] Umar E, Mandalazi P, Jere D, Muula A (2013). Should female health providers be involved in medical male circumcision? Narratives of newly circumcised men in Malawi. Malawi Med J.

[46] Douglas M, Hongoro C (2018). The consideration of socioeconomic determinants in prevention of traditional male circumcision deaths and complications. Am J Mens Health.

[47] Douglas M, Maluleke TX, Manyaapelo T, Pinkney-Atkinson V (2018). Opinions and perceptions regarding traditional male circumcision with related deaths and complications. Am J Mens Health.

[48] Prusente S, Khuzwayo N, Sikweyiya Y. Exploring factors influencing integration of traditional and medical male circumcision methods at Ingquza Hill local municipality, eastern cape: a socio-ecological perspective. Afr J Prim Health Care Fam Med 2019;11(1):1–1. doi: 10.4102/phcfm.v11i1.1948, e11. 10.4102/phcfm.v11i1.1948 PMC6739517 31478738

[49] Mahule A. Acceptability, Concerns and Experiences of Men Circumcised by Female Health Providers in Lusaka District [PhD Thesis]: The University of Zambia; 2016.

